# Astrocyte‐specific transcriptome analysis using the ALDH1L1 bacTRAP mouse reveals novel biomarkers of astrogliosis in response to neurotoxicity

**DOI:** 10.1111/jnc.14800

**Published:** 2019-07-11

**Authors:** Lindsay T. Michalovicz, Kimberly A. Kelly, Saurabh Vashishtha, Rotem Ben‐Hamo, Sol Efroni, Julie V. Miller, Alicia R. Locker, Kimberly Sullivan, Gordon Broderick, Diane B. Miller, James P. O’Callaghan

**Affiliations:** ^1^ Health Effects Laboratory Division, Centers for Disease Control and Prevention National Institute for Occupational Safety and Health Morgantown West Virginia USA; ^2^ Center for Clinical Systems Biology Rochester General Hospital Research Institute Rochester New York USA; ^3^ The Mina and Everard Goodman Faculty of Life Sciences Bar‐Ilan University Ramat‐Gan Israel; ^4^ School of Public Health Boston University Boston Massachusetts USA

**Keywords:** Astrocyte, astrogliosis, biomarker, neurotoxicity, transcriptome, translating RNA

## Abstract

Neurotoxicology is hampered by the inability to predict regional and cellular targets of toxicant‐induced damage. Evaluating astrogliosis overcomes this problem because reactive astrocytes highlight the location of toxicant‐induced damage. While enhanced expression of glial fibrillary acidic protein is a hallmark of astrogliosis, few other biomarkers have been identified. However, bacterial artificial chromosome ‐ translating ribosome affinity purification (bacTRAP) technology allows for characterization of the actively translating transcriptome of a particular cell type; use of this technology in aldehyde dehydrogenase 1 family member L1 (ALDH1L1) bacTRAP mice can identify genes selectively expressed in astrocytes. The aim of this study was to characterize additional biomarkers of neurotoxicity‐induced astrogliosis using ALDH1L1 bacTRAP mice. The known dopaminergic neurotoxicant 1‐methyl‐4‐phenyl‐1,2,3,6‐tetrahydropyridine (MPTP; 12.5 mg/kg s.c.) was used to induce astrogliosis. Striatal tissue was obtained 12, 24, and 48 h following exposure for the isolation of actively translating RNA. Subsequently, MPTP‐induced changes in this RNA pool were analyzed by microarray and 184 statistically significant, differentially expressed genes were identified. The dataset was interrogated by gene ontology, pathway, and co‐expression network analyses, which identified novel genes, as well as those with known immune and inflammatory functions. Using these analyses, we were directed to several genes associated with reactive astrocytes. Of these, TIMP1 and miR‐147 were identified as candidate biomarkers because of their robust increased expression following both MPTP and trimethyl tin exposures. Thus, we have demonstrated that bacTRAP can be used to identify new biomarkers of astrogliosis and aid in the characterization of astrocyte phenotypes induced by toxicant exposures.

**Open Science Badges:**

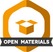

This article has received a badge for *Open Materials* because it provided all relevant information to reproduce the study in the manuscript. The complete Open Science Disclosure form for this article can be found at the end of the article. More information about the Open Practices badges can be found at https://cos.io/our-services/open-science-badges/.

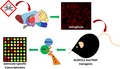

Cover Image for this issue: doi: 10.1111/jnc.14518.

Abbreviations usedALDH1L1: aldehyde dehydrogenase 1 family member L1
bacTRAP: bacterial artificial chromosome – translating ribosome affinity purification
CNS: central nervous system
DA: dopamine
DEG: differentially expressed gene
ELISA: enzyme‐linked immunsorbent assay
FACS: fluorescence‐activated cell sorting
FDR: false discovery rate
GED: graph edit distance
GFAP: glial fibrillary acidic protein
HPLC: high performance liquid chromatography
MPTP: 1‐methyl‐4‐phenyl‐1,2,3,6,‐tetrahydropyridine
NCI: National Cancer Institute
PFA: paraformaldehyde
PID: pathway interaction database
RRID: Research Resource Identifier (see scicrunch.org)
TH: tyrosine hydroxylase
TMT: trimethyl tin

Neurotoxic exposures damage diverse and unpredictable targets in the central nervous system (CNS). Astrogliosis ensues at the site of neurotoxic damage and can be quantified by assaying brain region‐specific levels of the astrocyte intermediate filament protein, glial fibrillary acidic protein (GFAP). Enhanced expression of GFAP is tightly linked to the damaged target, as well as the dose, time, and duration of the neurotoxic effect, regardless of the region or cell type affected (O’Callaghan and Sriram, 2005; O’Callaghan *et al.,* 2014). These observations suggest that astrogliosis is a common response to all types of neurotoxic exposures and that GFAP and other markers of ‘reactive’ astrocytes serve as biomarkers of neurotoxicity. Whether common or diverse signaling mechanisms underlie the conversion of astrocytes into their reactive state remains unknown. Discovering signaling mechanisms responsible for instigating astrogliosis would provide the earliest ‘signatures’ of all types of neurotoxicity and, potentially, would provide the basis for developing early therapeutic interventions through manipulation of astroglial reactivity. One strategy to discover and characterize astrocyte activation signaling is to identify gene expression patterns occurring only in reactive astrocytes as they respond to neural insults. Genomic analyses of astrogliosis in stroke and neuroinflammation models have been achieved through *in vitro* work with dissociated astrocytes prepared with fluorescence‐activated cell sorting (FACS) from the exposed mice (Zamanian *et al.,* 2012). The data obtained revealed striking subtypes of reactive astrocytes, however, these purified astrocytes may lack the influence of tissue‐intrinsic signals *in vivo*. A more direct approach to the analyses of gene expression events in reactive astrocytes, as they occur *in vivo*, may be needed to more fully characterize the molecular basis of astrogliosis.

The introduction of bacterial artificial chromosome – translating ribosome affinity purification (bacTRAP) technology to identify neural cell‐type‐specific responses *in vivo* (Doyle *et al.,* 2008) makes it possible to monitor *in vivo* gene expression events localized only to astrocytes. Monitoring astrocyte‐selective gene expression can be achieved through the use of aldehyde dehydrogenase 1 family member L1 (ALDH1L1) bacTRAP mice, as ALDH1L1 previously was identified as an astrocyte‐enriched gene (Dougherty *et al.,* 2010). Several studies have utilized the ALDH1L1 bacTRAP transgenic mice to evaluate different aspects of normal astrocyte gene expression, including regional or subcellular distribution and effects of the sleep–wake cycle (Bellesi *et al.,* 2015; Boulay *et al.,* 2017; Morel *et al.,* 2017), as well as gene expression changes related to temporal lobe epilepsy (Clasadonte *et al.,* 2016) and chronic stress (Simard *et al.,* 2018). However, these mice have not been used to evaluate astrogliosis related to neurotoxicity. Here, we began by using C57BL/6J and ALDH1L1 bacTRAP mice, respectively, to: (i) examine ALDH1L1 expression in astrocytes in response to neurotoxicant‐induced damage, and (ii) examine the effects of genotype on the neurotoxic response to a single dose of 1‐methyl‐4‐phenyl‐1,2,3,6‐tetrahydropyridine (MPTP). We then used ALDH1L1 bacTRAP mice to assess astrocyte‐localized gene expression in striatal reactive astrocytes responding to dopaminergic neurotoxicity resulting from the MPTP exposure. We found early and large fold changes in several astrocyte genes, implicating them in the conversion of astrocytes into their ‘reactive’ state following neurotoxicant exposure. Furthermore, additional analysis of these genes revealed that many had strong associations with known inflammatory and immune response pathways and that their expression changes were conserved across different neurotoxicant exposures, regardless of brain area or rodent species.

## Methods

### Materials

The following drugs and chemicals were provided by or obtained from the sources indicated: 1‐methyl‐4‐phenyl‐1,2,3,6‐tetrahydropyridine (MPTP; CAS# 23007‐85‐4) was purchased from Sigma‐Aldrich Co., (St. Louis, MO, USA); trimethyltin (TMT; CAS# 1066‐45‐1) was purchased from K&K Laboratories, Division of ICN Biochemical (Cleveland, OH, USA).

### Animals

All procedures were performed under protocols approved by the Institutional Animal Care and Use Committee of the Centers for Disease Control and Prevention, National Institute for Occupational Safety and Health (approved protocols 17‐016, 13‐JO‐M‐021, and 13‐JO‐R‐023), and the animal colony was accredited by AAALAC International. Adult male C57BL/6J (Cat# JAX:000664, RRID: IMSR_JAX:000664) mice (8–12 weeks of age; approximately 30 g) were purchased from Jackson Laboratories (Bar Harbor, ME, USA). Male Long–Evans rats (8 weeks of age; approximately 250g; Cat# 2308852, RRID: RGD_2308852) were purchased from Charles River Laboratories International, Inc. (Wilmington, MA, USA). ALDH1L1 bacTRAP founder animals were obtained from Jackson Laboratories (Cat# JAX:030247, RRID: IMSR_JAX:030247) and a colony was established at the Centers for Disease Control and Prevention, National Institute for Occupational Safety and Health by breeding heterozygous (+/−) males with heterozygous females. Each mouse in the litter was genotyped by tail biopsy using real‐time quantitative PCR of EGFP (5’‐CCTACGGCGTGCAGTGCTTCAGC; 3’‐CGGCGAGCTGCACGCTGCCGTCCTC), B‐actin (5’‐AGAGGGAAATCGTGCGTG AC; 3’‐CAATAGTGATGACCTGGCCGT), and Applied Biosystems CopyCaller (RRID:SCR_014486; Thermo Fisher Scientific, Waltham, MA, USA) software following the protocol available at bacTRAP.org (‘Protocol for Genotyping Mice’, www.bactrap.org/downloads.html). Both female and male mice were used for baseline comparisons of C57BL/6J, homozygous negative (−/−), heterozygous, and homozygous positive (+/+) carrying bacTRAP mice and ages ranged from 4 to 7 months. Mice and rats were arbitrarily housed individually in a temperature (21 ± 1°C) and humidity (50 ± 15%) controlled colony room maintained under filtered positive‐pressure ventilation on a 12‐h light/dark cycle beginning at 0600 Eastern Time. Rodents were given *ad libitum* access to food (Teklad 7913 irradiated NIH‐31 modified 6% rodent chow for mice and Teklad 2918 Global 18% for rats; Envigo, Madison, WI, USA) and water. Animals received daily health checks from animal husbandry personnel.

### Neurotoxicity models

**Figure 1:**
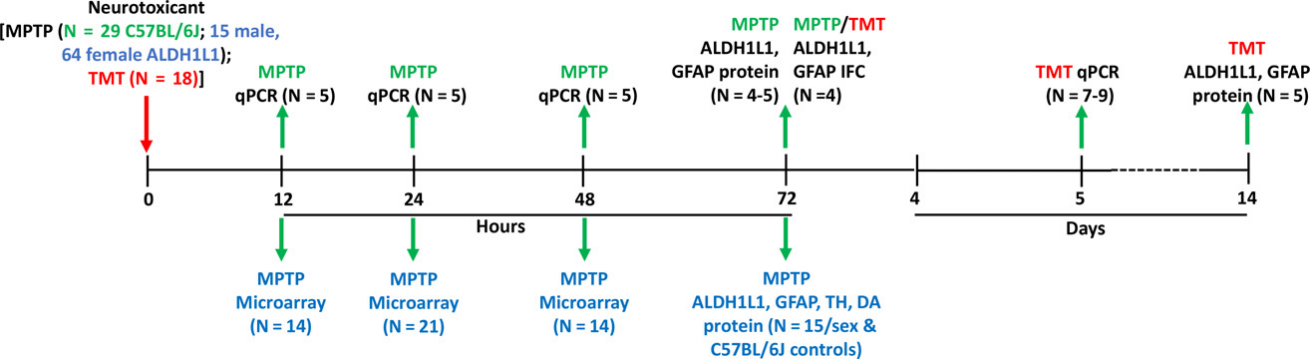
Experimental study design. Models of neurotoxicity, affecting both striatum [1‐methyl‐4‐phenyl‐1,2,3,6‐tetrahydropyridine (MPTP) in mice] and hippocampus [trimethyl tin (TMT) in rats], were employed in the study. The red arrow indicates the dosing of the neurotoxicant and green arrows indicate where samples were obtained for endpoint analysis. Assays indicated in green or blue text were performed following MPTP exposure in C57BL/6J or aldehyde dehydrogenase 1 family member L1, bacterial artificial chromosome ‐ translating ribosome affinity purification (ALDH1L1 bacTRAP) mice, respectively, while those indicated in red text were performed following TMT exposure in rats.

All animals were arbitrarily assigned to groups by the experimenter and identified by a number; the experimenter was not blinded to the groups and no randomization was performed to allocate subjects in the study. For detailed study design, see flowchart (Fig. 1). Neurotoxicants were administered in the morning to mice and rats in their home cages as follows: MPTP (12.5 mg/kg, s.c., single injection) male C57BL/6J mice (*N* = 29) and male (*N* = 15; 5/genotype)/female (*N* = 64; 5–12/genotype) ALDH1L1 bacTRAP mice; TMT (8 mg/kg, i.p., single injection) male Long–Evans rats (*N* = 18). No exclusion criteria were pre‐determined and no animals died during the experiments; in general, five animals were included per group to account for loss of at least one sample per endpoint to achieve a final *N* = 4 for statistical significance (see Methods: Statistical Analysis). Animals were killed by decapitation (protein and gene expression analysis) or terminal formalin perfusion (immunohistochemistry) beginning with the treated groups and followed by saline controls. In order to minimize risk of pain or suffering, all dosing and decapitations were performed by trained experimenters (as assessed by the Centers for Disease Control and Prevention, National Institute for Occupational Safety and Health Attending Veterinarian). Animals undergoing formalin perfusion were terminally anesthetized with Fatal Plus (NDC# 0298‐9373‐68, Vortech Pharmaceuticals, Dearborn, MI, USA) prior to the procedure. No additional medications or analgesics were given to the animals pre‐ or post‐dosing to reduce pain and/or suffering as this study only involved neuroactive compounds at doses that do not elicit pain.

### Protein immunoassay

For this analysis, animals (*N* = 5/group) were killed by decapitation 72 h post‐MPTP and 14 days post‐TMT. Whole brains were removed from decapitated rodents, striatum (after treatment with MPTP), or hippocampus (after treatment with TMT) were dissected free‐hand and homogenized with a sonic probe (model XL‐2005, Heat Systems, Framingdale, NY, USA) in 10 volumes of hot (90–95°C) 1% sodium dodecyl sulfate and stored at −80°C before assay of total protein, GFAP, tyrosine hydroxylase (TH), and ALDH1L1. Total homogenate protein was assayed using the Pierce^™^ BCA Protein Assay kit (Cat# 23225, Thermo Fisher Scientific), as per manufacturer’s instructions.

GFAP was measured by ELISA as described previously (Locker *et al*., 2017; O’Callaghan, 1991; O’Callaghan 2002). Briefly, GFAP protein from sodium dodecyl sulfate SDS homogenates was ‘sandwiched’ between rabbit polyclonal anti‐GFAP (1:400; Cat# Z0334, RRID: AB_10013382; DAKO, Carpenteria, CA, USA) and mouse monoclonal anti‐GFAP (1:250; Cat# G3893, RRID: AB_477010; Sigma‐Aldrich Co.) antibodies. An alkaline phosphatase‐conjugated antibody directed against mouse IgG (1 : 2000; Cat# 315‐055‐003, RRID: AB_2340075; Jackson ImmunoResearch Labs, West Grove, PA, USA) was then added and the colored reaction product was obtained using the enzyme substrate p‐nitrophenol. The ELISA was quantified by spectrophotometry (405 nm) on a Spectramax Plus 190 microplate reader (Molecular Devices, Sunnyvale, CA, USA) using Softmax Pro Data Acquisition and Analysis software (RRID:SCR_014240; Molecular Devices) and expressed as µg GFAP/mg total protein.

TH was also measured by ELISA as described previously (O’Callaghan *et al.,* 2014; Sriram *et al.*, 2004, 2006). Briefly, TH protein was ‘sandwiched’ between mouse monoclonal anti‐TH (1 : 500; Cat# T1299, RRID:AB_477560; Sigma‐Aldrich Co.) and rabbit polyclonal anti‐TH (1 : 500; Cat# 657012‐100UL, RRID:AB_696697; MilliporeSigma, Burlington, MA, USA) antibodies. An alkaline phosphatase‐conjugated antibody directed against rabbit IgG (1 : 3000; Cat# NA934, RRID:AB_772206; GE Healthcare, Chicago, IL, USA) was then added and the fluorescent substrate was obtained using Pierce™ Quantablu (Cat# 15169; Thermo Fisher Scientific). The ELISA was quantified by spectrophotometry (320/405 nm) as described above and expressed as µg TH/mg total protein.

ALDH1L1 was quantified using immunoblot analyses with detection of fluorescent signals using a LI‐COR Odyssey 9120 infrared fluorescence scanner (Lincoln, NE, USA). Immunoblots were obtained following resolution of total homogenate protein on 10% SDS‐PAGE gels, transferred to nitrocellulose membrane (Cat# GE10600000; MilliporeSigma), and blocked with Odyssey Blocking Buffer (Cat# 927‐40000, LI‐COR). Following incubation with primary antibody to ALDH1L1 (1 : 500, Cat# MABN495, RRID: AB_2687399; MilliporeSigma), blots were washed with phosphate‐buffered saline (PBS) containing 0.1% Tween 20 and incubated with fluorescent‐labeled secondary antibody (1 : 2500, Cat# 926‐68070, RRID: AB_10956588; LI‐COR) for 1 h prior to scanning by LI‐COR. A linear relationship between total protein load and signal intensity was confirmed as described previously (O’Callaghan *et al.,* 1999).

### Dopamine analysis

For this analysis, animals (*N* = 5/group) were killed by decapitation 72 h post‐exposure and striatum was dissected free‐hand from whole brains. Dopamine (DA) was analyzed by high performance liquid chromatography with electrochemical detection (HPLC‐EC), as described previously (Jones *et al.,* 2013). Briefly, frozen striatum were homogenized in ice‐cold 0.2 M perchloric acid, containing 1 um dihydroxybenzylamine as internal standard. Following centrifugation (10 000 g for 10 min), supernatants were filtered and aliquots (10 µL) were injected via a temperature‐controlled (4°C) automatic sample injector (Waters 717 plus autosampler, Waters Corporation, Milford, MA, USA) connected to a Waters 515 high performance liquid chromatography pump. DA was separated on a C18 reverse‐phase column (LC‐18 RP; Waters SYMMETRY, 25 cm × 4.6 mm; 5 µm) and electrochemically detected (Waters 464 Pulsed Electrochemical Detector) and analyzed with Waters Millennium software. Recovery of DA was adjusted with respect to the internal standard and quantified from a standard curve. The levels of dopamine were expressed as micrograms per gram of wet tissue.

### Immunofluorescence

For this analysis, animals were killed by decapitation 72 h post‐exposure. For double immunofluorescence colocalization analysis of ALDH1L1 (for mouse: 1 : 1000; Cat# ab87117, RRID: AB_10712968; Abcam, Cambridge, MA, USA; for rat: 1 : 500; Cat# MABN495, RRID: AB_2687399; Millipore Sigma) and GFAP (for mouse: 1 : 3000; Cat# 13‐0300, RRID: AB_2532994; Thermo Fisher Scientific; for rat: 1 : 2000; Cat# Z0334, RRID: AB_10013382; DAKO), animals (*N* = 4/group) were injected with a fatal dose of Fatal Plus and transcardially perfused with saline (0.9%) followed by 4% paraformaldehyde in 0.1M phosphate buffer, pH 7.4 paraformaldehyde to fix the brain tissue. Brains were removed and post‐fixed in paraformaldehyde for a minimum of 6 h. After post‐fixation, brains were transferred to 20% sucrose solution in 0.1 M phosphate buffer, pH 7.4 and shipped to FD Neurotechnologies (Columbia, MD, USA) for processing. The sections were visualized using an Olympus BX63 microscope (Center Valley, PA, USA) and images captured using Cell Sens Dimension software (v 1.15; RRID:SCR_016238**;** Olympus) with an Olympus DP73 digital camera attached to the microscope. Post‐processing of images was done according to accepted practices and image integrity guidelines (e.g., Cromey, 2010; Sedgewick, 2013).

### Gene expression analysis of TRAP‐ed astrocyte mRNA after MPTP

Female heterozygous ALDH1L1 bacTRAP mice were treated with saline (0.9%, s.c.) or MPTP (12.5 mg/kg, s.c.) and killed at 12, 24, or 48 h post‐injection. Brains were removed and striatum was dissected manually. Astrocyte‐specific RNA, bound to eGFP‐tagged polysomes, was isolated following the previously described protocol (Heiman *et al.,* 2014). Pooled striatal tissue (seven mice per sample; 2–3 pooled samples per group) from ALDH1L1 bacTRAP animals was homogenized and lysed to allow for immunoprecipitation of the eGFP‐tagged polysomes to anti‐eGFP‐bound (Cat# Htz‐GFP‐19F7, RRID:AB_2716736/Cat# Htz‐GFP‐19C8, RRID:AB_2716737, Memorial Sloan‐Kettering Monoclonal Antibody Facility, New York, NY, USA) Protein G magnetic beads (Dynabeads Cat# 10003D, Thermo Fisher Scientific). Beads were collected with a magnet (Dynabeads MPC‐S, Cat# A13346, Thermo Fisher Scientific) and the actively translating RNA was extracted and purified from the ALDH1L1‐eGFP cells using an RNeasy Mini kit per manufacturer’s instructions (Cat# 74104, Qiagen, Valencia, CA, USA).

Total RNA samples were sent to Q Squared Solutions Expression Analysis LLC (Morrisville, NC, USA) to determine the changes in actively translating RNA induced by MPTP neurotoxicity by Illumina MouseRef‐8 v2 Expression BeadChip (Cat# BD‐202‐0202, San Diego, CA, USA) microarray. Briefly, RNA integrity was confirmed prior to study and 100 ng of RNA was amplified and converted to biotin‐labeled cRNA. The BeadChips were scanned using an Illumina scan system and analyzed with Illumina GenomeStudio Analysis software (RRID: SCR_010973). A univariate analysis with one‐way anovas was used to find genes with a maxiumum log_2_ fold change ≥ 2 at least one time point with a false discovery rate (FDR) ≤ 0.01. This strategy identified 184 differentially expressed genes (DEGs) that were then analyzed across time with two‐way anova simple generalized linear model to reveal significantly increasing or decreasing expression by group over time following neurotoxic insult. Hierarchical clustering analysis was performed to evaluate the pattern of expression of the 184 DEG dataset across the 12, 24, and 48 h time points within each expression profile using ClustVis web tool (RRID:SCR_017133); time points were clustered using correlation distance and average linkage (Metsalu and Vilo, 2015). Gene ontology (GO) and KEGG Canonical pathway analysis of the 184 transcript set was performed using the Database for Annotation, Visualization and Integrated Discovery (DAVID, RRID: SCR_001881) (Dennis *et al.,* 2003). Of the 184 transcripts, 147 had available gene annotation. Terms from each ontology category (Molecular Function, Biological Process and Cellular Compartment) and pathways were sorted in DAVID based on the EASE *p*‐value (modified Fisher’s Exact test).

### Pathway activation analysis

To cast differences in transcript levels in a mechanistic context, we used the approach of Efroni et al. (Efroni *et al.,* 2007, 2008) to estimate the activity of known pathway segments from the expression of their component genes. The first step consists of converting a continuous measure of gene expression into a discrete gene status, namely up‐expressed or down‐expressed. In brief, for every individual gene the distribution of expression values across all samples are numerically fit to two separate gamma probability distribution functions: one describing the distribution of expression values supporting the up state and one describing the distribution of values supporting the down state. The probability of a gene being up‐expressed given its expression level *x* is the probability of occurrence of the ‘up’ state overall p_UP_ = N_UP_/N multiplied by the probability of expression level *x* corresponding to an up‐expressed state or γ(x;a_UP_, b_UP_); where N_UP_ is the number of genes that are up‐expressed among all N genes. It is important to note that this manipulation does not assign a discrete up or down state to a gene but instead provides a continuous scale of expression that includes being neither up‐ nor down‐expressed.

Every pathway consists of a collection of reaction steps or interactions. These can be modeled as logic functions with genes serving as inputs and outputs. In the current protocol, the activation level of such a logical function was computed based on the joint conditional probability that the input genes *k* ∈ set *I* are in an up‐expressed state based on measured gene expression (eqn (1)). As we have estimated the corresponding probability that the set of output genes *k* ∈ *O* are also in an up‐expressed state (eqn (2)), we can use the agreement between input and output as a measure of consistency *C*_s_ for the activation of reaction step *s* (eqn (3)).

(1) $$p\left(s=active\right)=\prod_{k\in I}p(g_{k}{=}^{\prime \prime }up^{\prime \prime })$$

(2) $$p\left(O{=}^{\prime \prime }up^{\prime \prime }\right)=\prod_{k\in O}p(g_{k}{=}^{\prime \prime }up^{\prime \prime })$$

(3) $$\begin{array}{c} C_{S}=p\left(s=active\right)\times p\left(O{=}^{\prime \prime }up^{\prime \prime }\right)+\left(1-p\left(s=active\right)\right)\times \left(1-p\left(O{=}^{\prime \prime }up^{\prime \prime }\right)\right) \end{array}$$

Using Equations (1), (2), (3), estimates of expected activation levels and the consistency of these estimates were calculated in each sample for all reaction steps described in 582 candidate pathways aggregated from the National Cancer Institute (NCI)‐Nature Pathway Interaction Database (PID, RRID: SCR_006866) (Schaefer *et al.,* 2009) and the Kyoto Encyclopedia of Genes and Genomes (KEGG, RRID: SCR_001120) database (Kanehisa *et al.,* 2010). The NCI‐Nature PID database is itself an aggregation of 135 pathways curated by the NCI‐Nature team with an additional 322 pathways imported from BioCarta (RRID:SCR_006917; www.biocarta.com) and Reactome (RRID: SCR_003485; Matthews *et al.,* 2009; Croft *et al.,* 2011). In each sample the activity of each pathway was calculated as the average activation level across all component interactions. Activation scores in each subject group were log transformed to improve normality and compared for each pathway at each time point using both parametric (*t* test) and non‐parametric (Wilcoxon rank sum) tests. Once again, two‐way analysis of variance (anova‐2) was used to assess the significance of group, time, and group x time interactions with the FDR estimated using Storey (Storey J.D., 2002). All the computations were conducted with the MATLAB software environment (RRID: SCR_001622; The MathWorks Inc., Natick, MA, USA). Significance in the overlap between the genes in the activated pathways with the full list of genes for the top five broader annotated canonical pathways was determined using a hypergeometric probability density function.

### Identification of co‐expression networks and network analysis

A simple linear correlation was used as a measure of association to describe the co‐expression of genes. It is important to note that the genetically identical mice were concatenated to incorporate perturbation and create a stronger basis for the correlation analysis because of the high degree of concordance between groups of control and exposed animals. Furthermore, we applied a leave‐one‐out subsampling strategy to the concatenated data and a mouse from each group, that is, exposed as well as unexposed, was left out in each subsample set. The resulting subsampled sets were used to compute correlation coefficients between gene pairs along with their respective null probability estimates. Null probability (p) was computed by transforming the correlation to create a t statistic having n‐2 degrees of freedom for n observations. Confidence bounds were based on an asymptotic normal distribution of 0.5*log ((*1 + r (x_i_ x_j_ | x_k_*))*/*(*1‐r (x_i_ x_j_ | x_k_*))), with an approximate variance equal to 1/(*n*‐3) when variables have a multivariate normal distribution. As there exist 16 836 (*n* (*n*‐1)/2) possible pair‐wise interactions in a 184‐node network, null probability values were adjusted for multiple comparisons using Storey’s method (Storey, 2002) for estimating the adjusted *p*‐ values (*q*‐static). Furthermore, correlation networks for each group were generated with a 100% consensus across subsampled networks to ensure the robustness of consensus network. More precisely, only edges that were assigned a significant correlation at a low q value (*q* < 0.05) in networks for each subsample were included in the consensus co‐expression networks.

Response networks were compared to evaluate general topological differences in overall structure of networks over time using a generalized measure of graph edit distance (GED) for continuously weighted graphs (Dickinson *et al.,* 2004). The GED was calculated by summing up the minimum ‘cost’ occurred in removing and inserting graph edges to transform one network into another. The distance implied is proportional to the magnitude of the weighted change in each edge. The weighted GED, dGED, between two undirected networks of order N with adjacency matrices, A and B, can be described as follows:

(4) $$d_{\mathrm{GED}}=\sum_{i=1}^{N}\sum_{j⩾i}^{N}|A_{\mathrm{ij}}-B_{\mathrm{ij}}|$$

The null probabilities of significance of differences in GED were calculated by comparing the GEDs of different subsampled networks.

In addition to the changes in overall structure of the network, local changes at the level of each node are also of significance since accumulation of local changes derives these global topological differences. Median node centrality measures such as degree centrality, betweenness centrality, and closeness centrality are also reported to describe the local changes in the networks around each node. Moreover, we also reported the importance of nodes in terms of hubs and authorities in the networks. Further details about these node centrality measures can be seen in Supporting Information Methods.

### Quantitative PCR (qPCR) analysis

For this analysis, mice and rats (*N* = 4–9/group) were killed post‐dosing as follows: MPTP (12, 24, 48 h) and TMT (5 days). Total RNA from striatum (MPTP) and hippocampus (TMT) was isolated as previously described (Locker *et al.*, 2017; Kelly *et al.*, 2018). Real‐time PCR analysis of the housekeeping gene, GAPDH (Cat# 4331182, Mm99999915_g1/Rn01775763_g1), and target genes: TIMP1 (Cat# 4331182, Mm01341361_m1/Rn01430873_g1), SERPINF2 (Cat# 4331182, Mm00435868_m1/Rn01464595_m1), AA467197/miR‐147 (Cat# 4351372, Mm01268692_m1), SOCS3 (Cat# 4331182, Mm00545913_s1/Rn00585674_s1), ADAMTS1 (Cat# 4351372, Mm01244169_m1/Cat# 4331182, Rn00577887_m1), GLDN (Cat# 4331182, Mm00616548_m1/Rn00710589_m1), NES (Cat# 4331182, Mm00450205_m1/ Cat# 4351372, Rn01455599_g1), ECM1 (Cat# 4331182, Mm00514634_m1/Rn01534170_g1), and FXYD5 (Cat# 4331182, Mm00435435_m1/Rn00573226_m1) was performed using FAM‐ MGB TaqMan® primers from Thermo Fisher and an ABI7500 Real‐Time PCR System (Thermo Fisher Scientific) in combination with TaqMan® chemistry as previously described (Locker *et al.*, 2017; Kelly *et al.*, 2018). Relative quantification of gene expression was performed using the comparative threshold (ΔΔC_T_) method. Changes in mRNA expression levels were calculated after normalization to GAPDH. The ratios obtained after normalization are expressed as fold change over corresponding saline‐treated controls.

### Statistical analysis

For calculation of sample size, one‐way anova sample size analysis was performed using SigmaPlot (v12.5, RRID: SCR_003210; Systat Software, Inc, San Jose, CA, USA) using the measures defined by the test: previously obtained minimum detectable difference [largest measured saline value (0.1 µg GFAP/mg total protein) versus smallest measured 72 h post‐MPTP value (0.446 µg GFAP/mg total protein); 0.346 µg GFAP/mg total protein] and largest measured standard deviation (0.14) in striatal GFAP protein (as measured by GFAP ELISA) between MPTP‐treated and control tissue (group size = 2) from C57BL/6J mice with a power of 0.8 and α = 0.05; the sample size was estimated at four mice per group for gene and protein expression analyses. Datasets were evaluated by Grubbs’ test (α = 0.05) and outliers were excluded from analyses. Sample size for microarray analysis was based on recommendations from the service providers. Generally, one or two‐way anovas were conducted to determine group differences using SigmaPlot statistical software. Specifically, two‐way anovas with Shapiro–Wilk normality test were performed on log transformed raw data for protein analyses in ALDH1L1 bacTRAP mice (Figs 4 and 5). Data were log transformed as they did not otherwise follow a normal distribution. One‐way anovas were performed using group mean, N, and SEM. Values were considered statistically significant at *p* < 0.05 using Fisher least significant difference post‐hoc analysis following anovas. Data are graphed as mean ± SEM with overlay of individual data points. For gene ontology annotation, canonical pathway, activated pathway, and co‐expression network analyses, see sections above for detailed statistics. Microarray data is available through Mendeley Data (https://doi.org/10.17632/ktgcp4mtk2.1); all other data are available from corresponding author upon request. These studies were not pre‐registered.

## Results

### ALDH1L1 levels are unaffected across different models of neurotoxicity

**Figure 2:**
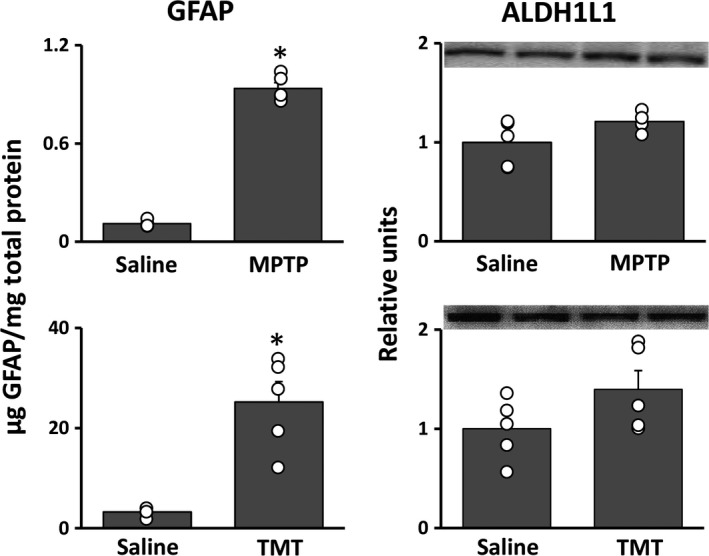
ALDH1L1 protein concentration is unaffected by neurotoxicant induced astrocyte hypertrophy. The striatal neurotoxicant 1‐methyl‐4‐phenyl‐1,2,3,6‐tetrahydropyridine (MPTP) and hippocampal neurotoxicant trimethyl tin (TMT) were employed to compare the aldehyde dehydrogenase 1 family member L1 (ALDH1L1) protein concentration in control and astrocyte hypertrophy conditions. Glial fibrillary acidic protein (GFAP) protein concentration was also measured as a positive control for astrocyte hypertrophy. All bars represent mean ± SEM (*N* = 4–5 animals/group) with overlay of individual data points; representative blots for ALDH1L1 are shown (*N* = 2 animals/group). Statistical significance was measured by one‐way anova with Fisher’s least significant difference method of post‐hoc analysis. Statistical significance of at least *p* < 0.05 for the neurotoxicant exposed groups in comparison to saline controls is denoted by *.

**Figure 3:**
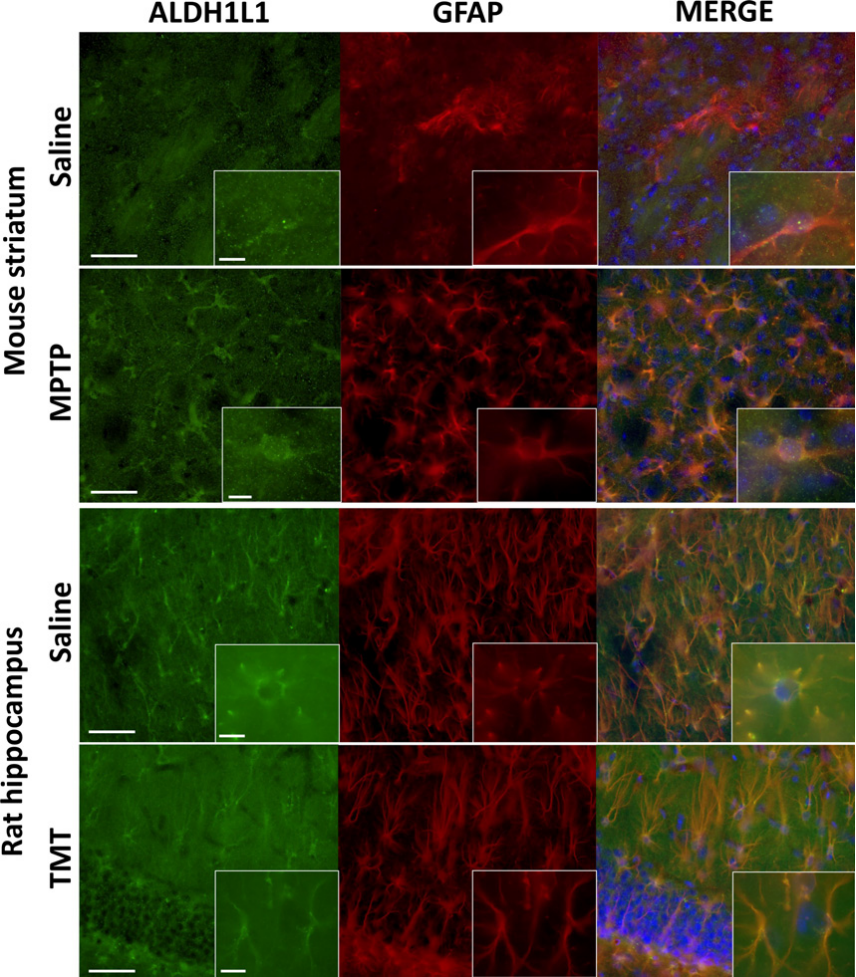
Colocalization of ALDH1L1 and GFAP in striatum and hippocampus. C57BL/6J mice exposed to 1‐methyl‐4‐phenyl‐1,2,3,6‐tetrahydropyridine (MPTP) or Long‐Evans rats exposed to trimethyl tin (TMT) were processed 72 h after exposure for immunohistochemical analyses of glial fibrillary acidic protein (GFAP) to identify astrocytes and their colocalization with aldehyde dehydrogenase 1 family member L1 (ALDH1L1). Merge of GFAP and ALDH1L1 is shown with DAPI for clarity of nucleus localization. Scale bars = 50 µm for 20× and 10 µm for 100× insets.

Astrogliosis, the reactive state of astrocytes, is the hallmark of all types of CNS injuries and damage, and enhanced expression of the intermediate filament protein of astrocytes, GFAP, is the characteristic feature. ALDH1L1 has been reported to be localized to astrocytes (Neymeyer *et al.,* 1997; Cahoy *et al.,* 2008; Doyle *et al.,* 2008; Dougherty *et al.,* 2010). To determine if ALDH1L1, like GFAP, is up‐regulated in response to neural damage, we administered MPTP and TMT to compare the protein expression of GFAP with that of ALDH1L1 in the brain regions targeted by these compounds. The known striatal dopaminergic nerve terminal neurotoxicant, MPTP, caused large increases (> 4‐fold) in striatal levels of GFAP (Fig. 2). Levels of ALDH1L1 in the same tissue samples were not affected. The known hippocampal neurotoxicant, TMT, also caused increases in hippocampal levels of GFAP without affecting ALDH1L1 levels. These observations were further verified by ALDH1L1 and GFAP co‐immunofluorescence staining of histological brain sections from mice treated with MPTP and rats treated with TMT (Fig. 3). Not only does ALDH1L1 colocalize to GFAP + astrocytes in both the striatum and hippocampus, but histological evaluation following MPTP exposure confirms the measured increase in GFAP expression as well as the morphological hypertrophy and increased number of activated astrocytes associated with astrogliosis following exposure. As a result of the density of astrocytes in the hippocampus, these morphological changes are harder to discern histologically following TMT exposure. These findings replicate past observations for the effects of these neurotoxicants on GFAP (e.g., O’Callaghan *et al.,* 2014) and suggest that ALDH1L1 is more stably expressed in astrocytes by comparison.

### ALDH1L1 bacTRAP genotype affects GFAP, TH, and DA response to MPTP‐induced neurotoxicity

To begin to assess the utility of ALDH1L1 bacTRAP mice to explore gene expression in reactive astrocytes, we evaluated their general health by looking at the influence of ALDH1L1 genotype on body, organ, and brain weights (Supporting Information Table S1). While the location of the BAC insertion does not seem to affect a given bacTRAP mouse phenotype (Doyle *et al.,* 2008), the possibility remains that genotype may have unintended consequences (e.g., act as a mutation, alter response to external factors). After administration of MPTP to male and female mice, body, organ, total brain, and brain regional weights were not affected by genotype in comparison to strain controls or wild‐type C57BL/6J mice (Supporting Information Table S1).

**Figure 4:**
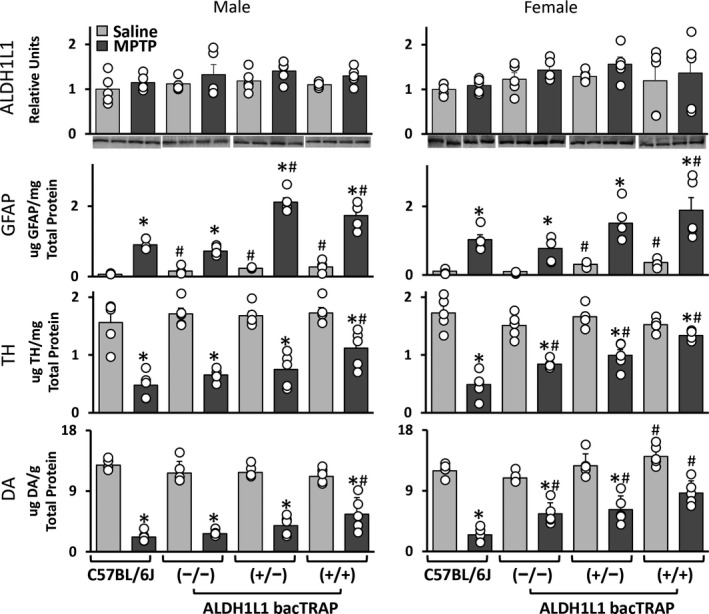
Effects of the ALDH1L1 bacTRAP transgene on GFAP expression and dopaminergic neuron damage following MPTP. C57BL/6J control mice and aldehyde dehydrogenase 1 family member L1, bacterial artificial chromosome ‐ translating ribosome affinity purification (ALDH1L1 bacTRAP) −/−, +/−, and +/+ mice were treated with saline or 1‐methyl‐4‐phenyl‐1,2,3,6‐tetrahydropyridine (MPTP). ALDH1L1, glial fibrillary acidic protein (GFAP), tyrosine hydroxylase (TH), and dopamine (DA) protein concentration were measured in striatum at 72 h post‐exposure. All bars represent mean ± SEM (*N* = 4‐5 mice/group) with overlay of individual data points; representative blots for ALDH1L1 are shown (*N* = 2 animals/group). Statistical significance of at least *p* < 0.05 for the neurotoxicant alone groups compared to saline control (*) and C57BL/6J (#).

We then determined if genotype influenced neuronal and astrocytic responses to neurotoxic insult with the known dopaminergic neurotoxicant, MPTP. ALDH1L1 protein concentration between C57BL/6J, homozygous negative (−/−), heterozygous (+/−), and homozygous positive (+/+) bacTRAP mice showed no significant differences in either male or female mice under both saline and MPTP exposure conditions (Fig. 4). When we assessed baseline striatal levels of GFAP, saline‐treated +/− and +/+ bacTRAP mice demonstrated enhanced expression of this protein compared to C57BL/6J mice, regardless of sex (Fig. 4). Furthermore, there were genotype differences in MPTP‐induced GFAP levels for +/− and +/+ bacTRAP males, as well as +/+ bacTRAP females, which had exacerbated GFAP responses compared to C57BL/6J (Fig. 4). For both male and female bacTRAP mice, the degree of dopaminergic nerve terminal damage in response to MPTP exposure was less severe compared to C57BL/6J or −/− bacTRAP mice, as indicated by higher levels of tyrosine hydroxylase protein and higher levels of dopamine. This effect was most pronounced in female +/− and +/+ bacTRAP mice (Fig. 4). Specifically, baseline dopamine levels in female +/+ bacTRAP mice were significantly higher than their C57BL/6J counterparts. Moreover, when these mice were treated with MPTP, they did not exhibit a significant reduction in dopamine concentration (Fig. 4). Interestingly, evaluation of naïve (non‐handled) bacTRAP males and females, based upon genotype alone, indicated no statistically significant group differences in dopamine or serotonin metabolites (data not shown). These data may suggest that ALDH1L1 bacTRAP mice mount a larger astrogliotic response to a given level of dopaminergic neurotoxicity caused by MPTP. However, it is also possible that as an intermediate filament that contributes to the structure of the astrocyte, an increase in GFAP could mean that the astrocytes in +/− and +/+ bacTRAP mice are simply larger than those in −/− bacTRAP or C57BL/6J mice.

### Astrocyte gene‐expression profile after MPTP in ALDH1L1 bacTRAP mice

Astrogliosis, as evidenced by enhanced expression of GFAP, is initiated 12 h after MPTP‐induced neurotoxicity to striatal dopaminergic nerve terminals, with peak astrogliosis reached by 72 h post‐dosing followed by rapid declines to near control values by 2 weeks (O’Callaghan *et al.,* 1990; Sriram *et al.,* 2004; O’Callaghan *et al.,* 2014). Based on these time course data and the smaller observed effects on GFAP (Fig. 4), we evaluated TRAP‐ed striatal astrocytic gene expression profiles in female +/− ALDH1L1 bacTRAP mice at 12, 24, and 48 h after administration of MPTP to capture effects at the outset and early stages of astrogliosis.

**Figure 5:**
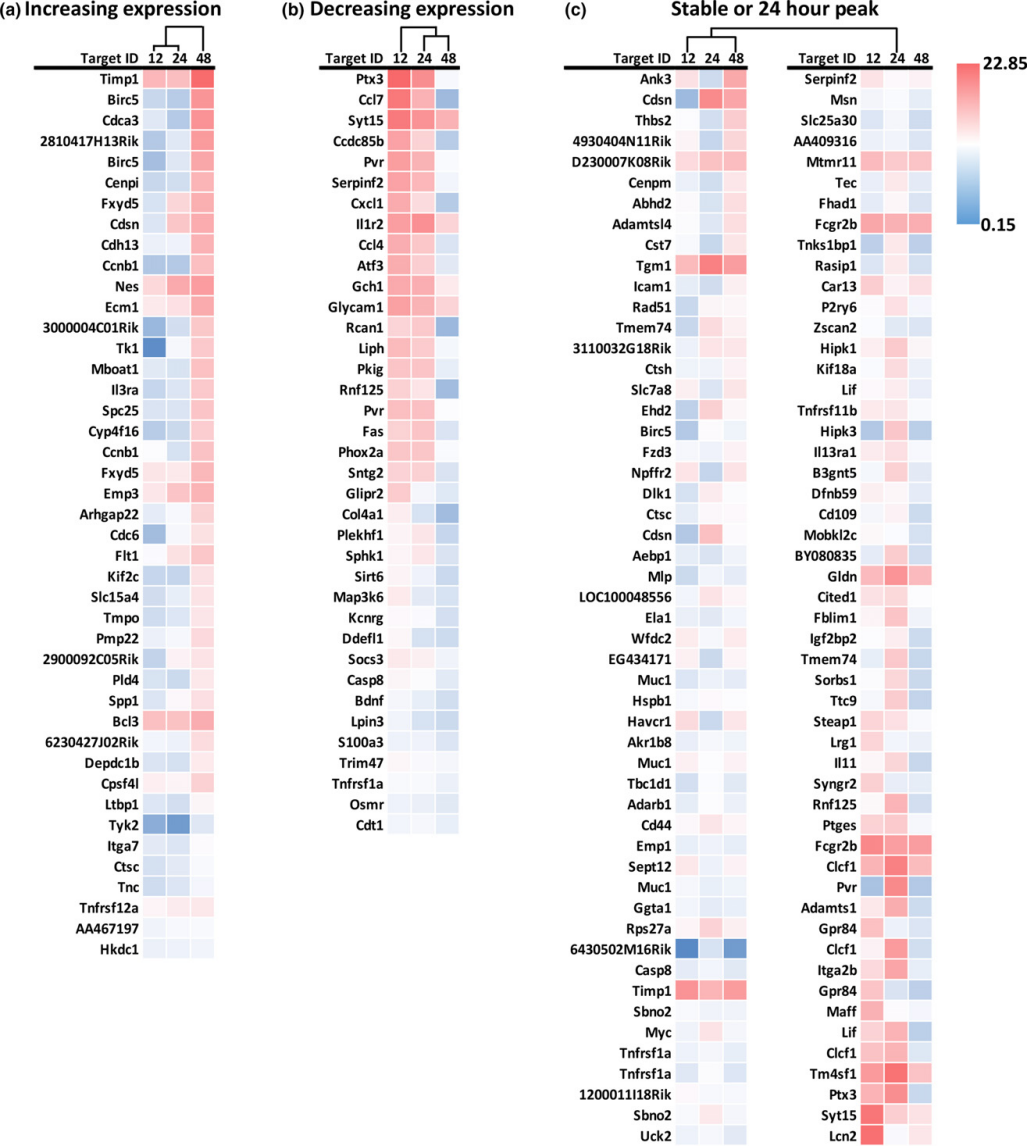
Heat map profile of astrocyte gene expression after MPTP exposure in ALDH1L1 bacTRAP mice. Female aldehyde dehydrogenase 1 family member L1, bacterial artificial chromosome ‐ translating ribosome affinity purification (ALDH1L1 bacTRAP) +/− mice were treated with saline or 1‐methyl‐4‐phenyl‐1,2,3,6‐tetrahydropyridine (MPTP). Microarray analysis was performed on pooled striatal tissue (*N* = 7 mice/sample, 2–3 samples/group) collected at 12, 24, and 48 h post‐exposure. Univariate analysis identified 184 statistically significant, differentially expressed genes (DEGs) with a maxiumum log_2_ fold change ≥ 2 at least one time point with a FDR ≤ 0.01. Differentially expressed genes were separated and sorted based on their slope indicating increasing expression over time (a), decreasing expression over time (b), or those genes having peak expression at 24 h or a stable increased or decreased expression over time (c). These individual datasets were then subjected to hierarchical cluster analysis to determine similarities between the expression profiles across the three time points.

Univariate analysis of the 16 193 BeadChip gene probes revealed 184 DEGs where the MPTP‐induced log_2_ fold change was at least 2 at 12, 24, or 48 h after exposure with a FDR < 0.01. Subsequent linear correlation analysis revealed significant change across the time course with 43 genes increasing and 37 genes decreasing during the 12‐ to 48‐hour time window, with the remaining genes having a peak at 24 h or being stably altered compared to controls (Fig. 5). Hierarchical cluster analysis to compare the expression pattern across the three time points revealed unique clustering patterns for each grouping of genes (Fig. 5). For those genes that increased over time, the 12‐ and 24‐hour expression profiles were very similar (Fig. 5a). However, the expression profiles were more greatly dissimilar at each time point for the DEGs that decreased over time (Fig. 5b) and those that exhibited a peak at 24 h or were stable altered over time (Fig. 5c). Furthermore, results of a two‐way anova indicated that in 40 of these 80 genes the trajectory with time observed in MPTP exposed animals diverged significantly with that observed in unexposed animals (time × group effect FDR < 0.01). We then subjected the 184 DEGs to gene ontology (Table 1) and canonical pathway (Table 2) analyses. Immune‐ and cytokine‐related functions predominated at the molecular and process levels and many immune signaling‐related extracellular matrix components were dominant at the cellular level (Table 1), highlighting the role of astrocytes in inflammatory signaling and likely indicating changes in morphology and cell–astrocyte interactions following activation of the astrocyte. The focus on immune function was further highlighted by the canonical pathway analysis with cytokine receptor signaling and downstream signaling cascades (Jak‐STAT and PI3K‐Akt) being the most significantly affected canonical pathways after MPTP exposure (Table 2).

**Table 1:** Gene ontology categorization of DEGs identified in astrocytes post‐1‐methyl‐4‐phenyl‐1,2,3,6,‐tetrahydropyridine (MPTP) exposure

	Term^a^	*p*‐value^b^	DEGs identified within GO term
Molecular Function	protease binding	2.23E‐05	LCN2, TNFRSF1A, SERPINF2, CST7, ADAMTSL4, FAS, ECM1, TIMP1
	cytokine activity	9.32E‐04	CXCL1, LIF, CLCF1, CCL4, CCL7, SPP1, TIMP1, IL11
	identical protein binding	1.90E‐03	FLT1, BIRC5, KCNRG, CCL4, RAD51, TK1, ATF3, CASP8, HSPB1, CTSC, FAS, PTX3, ITGA2B
	peptidase inhibitor activity	1.99E‐03	SERPINF2, CST7, CD109, BIRC5, WFDC2, TIMP1
	cytokine receptor activity	4.18E‐03	CD44, OSMR, IL13RA1, IL3RA
Biological Process	negative regulation of peptidase activity	2.18E‐04	SERPINF2, CST7, CD109, BIRC5, ECM1, WFDC2, TIMP1
	positive regulation of angiogenesis	2.61E‐04	TNFRSF1A, FLT1, LRG1, SPHK1, HSPB1, ECM1, CTSH
	response to lipopolysaccharide	6.13E‐04	CXCL1, TNFRSF1A, TNFRSF11B, PTGES, CASP8, FAS, CITED1, GCH1
	positive regulation of fibroblast proliferation	1.30E‐03	CCNB1, CDC6, SPHK1, SIRT6, MYC
	positive regulation of cell proliferation	2.09E‐03	LIF, ATF3, HIPK1, OSMR, CLCF1, TNC, SPHK1, RASIP1, MYC, CTSH, TIMP1, IL11
Cellular Compartment	extracellular space	1.30E‐08	CXCL1, AEBP1, LTBP1, GLDN, TNC, CD109, DLK1, CCL4, CCL7, TIMP1, IL11, LIF, TNFRSF1A, TNFRSF11B, BDNF, LRG1, CLCF1, MSN, FAS, PTX3, RPS27A, SPP1, ICAM1, FLT1, ECM1, LCN2, CDH13, CST7, SERPINF2, HSPB1, LIPH, CTSC, CTSH
	extracellular region	1.39E‐06	CXCL1, IL1R2, AEBP1, LTBP1, GLDN, TNC, ADAMTSL4, CD109, CDSN, CCL4, CCL7, TIMP1, IL11, LIF, GLIPR2, TNFRSF11B, BDNF, GLYCAM1, CLCF1, FAS, PTX3, THBS2, SPP1, COL4A1, ECM1, LCN2, FCGR2B, CST7, SERPINF2, LIPH, ADAMTS1, WFDC2
	cell surface	1.51E‐04	MUC1, ICAM1, HAVCR1, TNFRSF12A, CD109, FZD3, TNFRSF1A, CD44, FCGR2B, SERPINF2, ANK3, ITGA7, MSN, FAS, ITGA2B
	extracellular matrix	2.48E‐04	AEBP1, COL4A1, LTBP1, ADAMTSL4, TNC, HSPB1, ADAMTS1, ECM1, THBS2, TIMP1
	perinuclear region of cytoplasm	4.29E‐04	LTBP1, RASIP1, 2810417H13RIK, RAD51, SEPT12, PLEKHF1, CDH13, BDNF, PTGES, BCL3, FAS, MSN, EHD2, MYC, SPP1

a The top five significant terms are shown for each ontology domain.

b Indicates the significance of the representation (enrichment) of the term within the DEG dataset.

**Table 2:** Canonical pathway classification of DEGs identified in astrocytes post‐1‐methyl‐4‐phenyl‐1,2,3,6,‐tetrahydropyridine (MPTP) exposure

Pathway name^a^	*p*‐value^b^	Genes
Cytokine–cytokine receptor interaction	2.72E‐07	IL1R2, FLT1, TNFRSF12A, OSMR, CCL4, CCL7, IL11, LIF, TNFRSF1A, TNFRSF11B, CLCF1, FAS, IL13RA1, IL3RA
TNF signaling pathway	5.83E‐05	CXCL1, LIF, ICAM1, TNFRSF1A, SOCS3, CASP8, BCL3, FAS
ECM‐receptor interaction	1.46E‐04	COL4A1, CD44, TNC, ITGA7, THBS2, ITGA2B, SPP1
Jak‐STAT signaling pathway	3.48E‐04	TYK2, LIF, OSMR, SOCS3, IL13RA1, MYC, IL3RA, IL11
PI3K‐Akt signaling pathway	4.61E‐03	COL4A1, FLT1, OSMR, TNC, ITGA7, MYC, THBS2, IL3RA, ITGA2B, SPP1

a The top five significant pathways are shown.

b Indicates the significance of the association (enrichment) of the DEG dataset with the canonical pathway.

### Signaling Pathway Activation Analysis by MPTP exposure reveals involvement of several cytokine pathways

Canonical pathways tend to include very large and complex signaling pathways that can extend across multiple cell types and organs, making it difficult to interpret the significance of these immense signaling cascades. By estimating the activation state of more discrete signaling pathways, we attempted to gain a more insightful grasp of the astrocytic pathways activated by MPTP exposure in the ALDH1L1 bacTRAP mice. An analysis of predicted pathway activation levels based upon gene ‘up’ or ‘down’‐expression suggested that 15 of the 588 known pathways documented in the NCI‐Nature PID displayed a fold change in activation greater than 1.5 across all three time points at a FDR < 0.05 (Table 3). Of these, 12 pathways showed a divergent trajectory across time between MPTP‐exposed and unexposed animals (time × group effect FDR < 0.05). Among these pathways, we found that regulation of p38 alpha and beta MAP kinase signaling, as well as its co‐regulation with cdk5/p35 signaling, was a dominant theme (Table 3). This MAP kinase signaling is central to the induction of the nitric oxide response and associated angiogenesis in an inflammatory environment (Da Silva *et al.,* 1997). Not surprisingly, a number of specific cytokine signaling pathways were activated in astrocytes following exposure to MPTP including GATA3 activation of Th2 cytokine expression and IL‐10 anti‐inflammatory pathway activation with corresponding modulation of inflammatory signaling involving, for example, broad‐acting IFNγ (Miljkovic *et al.,* 2007). Specifically, IL‐4 mediated signaling was consistently modulated at 12 and 24 h in MPTP‐treated animals only. Consistent with this environment, we also find increased modulation of integrin signaling (a4b4, a4b7) and their role in leukocyte adhesion.

**Table 3:** Pathways activated in astrocytes in 1‐methyl‐4‐phenyl‐1,2,3,6,‐tetrahydropyridine (MPTP) exposed mice

	Fold change^a^				
Pathway name	12 h	24 h	48 h	Overlap^b^	Genes
epha2 forward signaling (nci/nature)	1.80	1.98	0.86	5	ARHGAP35, PAK1, FAK1, CBL, SHIP2, SHC1, CA1, ACP1, SRC, EFNA1, EPHA2, TIAM1, VAV2, VAV3, GRB2
gata3 participate in activating the th2 cytokine genes expression (biocarta)	2.04	2.00	1.00	1,2,4	PKA, IL4, JUNB, NFATc1, MAF, GATA3, MAPK14, MAP2K3, IL13, MAP2K6, IL5
human cytomegalovirus and map kinase pathways (biocarta)	1.60	1.65	0.84	2,5	CREB1, MAP3K1, MAP2K1, MAP2K2, MAPK14, PDPK1, SP1, MAPK1, MAPK3, AKT1, MAP2K6, MAP2K3, RB1
ifn‐gamma pathway (nci/nature)	2.35	1.97	1.19	2,4	PIAS1, CAMKII family, CBL, SMAD7, PKCD, MEKK1, EP300, CBP, STAT1, IRF1, IL1B, CASP1, MEK1, PTPN2, DAPK1, SOCS1, CEBPB, MAP3K11, ERK1, ERK2, SHP2, CALM1, mTOR, AKT1, IRF9, TC45, PIASy, PTGES2
il‐10 anti‐inflammatory signaling pathway (biocarta)	1.60	1.57	1.00	1,2,4	IL6, MAPK14, MAP2K6, HMOX1, MAP3K5, IL10, IL1A, BLVRA, TNF
il4‐mediated signaling events (nci/nature)^c^	Inf	Inf	NaN	1,2,3,4,5	ALOX15, SOCS1, IL5, SOCS5, HMGIY, BCL6, CEBPB, IgE, TFF3, IRS2, mTOR, MAPK14, SOCS3, CBL, IL10, IgHG3, FES, SHP1, CCL17, ARG1, SPI1, CCL11, GRB2, STAT6, COL1A2, SP1, IRS1, EGR2, IL4, SHIP, cd61, COL1A1, GTF3A, ETS1, Myb, PSEL, TNFB, BCL2L1, SHC, AID, PIGR, THY1, DOK2, IRF4, CD40LG, FIZZ1, AKT1, IL4R, IL13RA2, CCL26, FCER2, STAT5, p70S6K, IgHG4, OPRM1
p38 mapk signaling pathway (biocarta)	1.66	1.61	1.07	2,5	DDIT3, MAPK14, PLA2G1B, HMGN1, RPS6KA5, MAX, ELK1, MYC, MAP2K4, MAP3K9, MAPKAPK5, CREB1, H3F3A, H3F3B, MEF2, MKNK1, RAC1, HRAS, MAP3K1, MAP2K6, NR2C2, HSPB1, CDC42, MAP3K5, ATF2, MAPKAPK2, STAT1
paxillin‐independent events mediated by a4b1 and a4b7 (nci/nature)	494.49	115.10	0.24	3	FAK1, JAK2, CAS1, DOCK1, CRK, SRC, CD44, VCAM1, ARF6, RHOA, MADCAM1
phosphorylation of mek1 by cdk5/p35 down‐regulates the map kinase pathway (biocarta)	2.79	1.76	1.33	2,5	MAP2K1, RAF1, MAP2K2, EGR1, MAPK1, MAPK3, HRAS, CDK5R1, CDK5, NGFB, NGFR
regulation of p38‐alpha and p38‐beta (nci/nature)	19.20	14.75	1.57	2	MAPK14, SRC, ATF2, JNK, TAB1, MKP1, MKP7, MKP5, RIP1, MEKK3, DUSP8, MKK3, PAK1, MAPK11, MKK6, DLK, TRAF6, MKK4
signaling mediated by p38‐alpha and p38‐beta (nci/nature)	6.52	6.70	0.98	2,5	CHOP, MAPK14, ATF2, MEK3, MEK6, ATF6, PRAK, MSK1, MSK2, SAP1, PGES2, USF1, HBP1, PLA2G4A, P53, MNK1, GDI1, NOS2, MEF2A, EIF4E, MITF, MAPKAPK3, PGC1, CREB1, CEBPB, KRT8, NHE1, ESR1, MAPKAPK2, MEF2C, ATF1, HSP27, KRT19, EIF4EBP1
the 41bb‐dependent immune response (biocarta)^d^	Inf	Inf	NaN	1,2,4,5	JUN, IL2, MAPK14, PKC, MAP3K5, IFNG, MAPK14, TNFSF9, IL4
transcription factor creb and its extracellular signals (biocarta)	1.53	1.56	1.08	2,5	MSK1, PDPK1, JUN, GZMA, AKT1, RPS6KA1, HRAS, ERK1, ERK2, CAM
tsp‐1 induced apoptosis in microvascular endothelial cell (biocarta)^d^	Inf	Inf	NaN	2,3	MAPK14, FOS, ERK1, ERK2, PKC, CASP3
vegfr3 signaling in lymphatic endothelium (nci/nature)	1.50	1.49	1.00	2,5	MKK4, ERK1, ERK2, VEGFR3, CRK, CREB1, RPS6KA1, MAPK11, VEGFC, VEGFD, AKT1, MAPK14

a FC defined as mean (Act treated)/ mean (Act untreated).

b Defined as significant (*p* < 0.05) overlap of pathway components with canonical pathways, where: 1, Cytokine–cytokine receptor interact; 2,TNF signalling; 3, ECM‐receptor interact; 4, Jak‐STAT signaling; 5, PI3K‐Akt signaling.

c Pathway is not consistently expressed in untreated and at 48h in treated.

d Pathways are not active in untreated.

Our evaluation of the overlap between the individual canonical and activated pathways gene sets revealed that all of the activated pathways shared a significant number of genes with canonical pathways (Table 3), thus representing the vast inclusiveness of the canonical pathways. The activated pathways overlapped most frequently (14 times) with the TNF signaling pathway, suggesting that this particular pathway may be activated on many levels; not a surprising finding considering the prominence of inflammatory signaling amongst the pathways and the role of TNF as a major cytokine signaling hub (Chen and Goeddel, 2002; Sriram and O'Callaghan 2007; Bradley 2008; Wallach 2016). Interestingly, all of the canonical pathways significantly overlapped with the IL‐4 mediated signaling pathway indicating that these different canonical pathways may be significant in toxicant‐induced astrogliosis because of their convergence on this signaling module.

### Co‐expression network analysis identified several unique transcripts with high centrality

**Figure 6:**
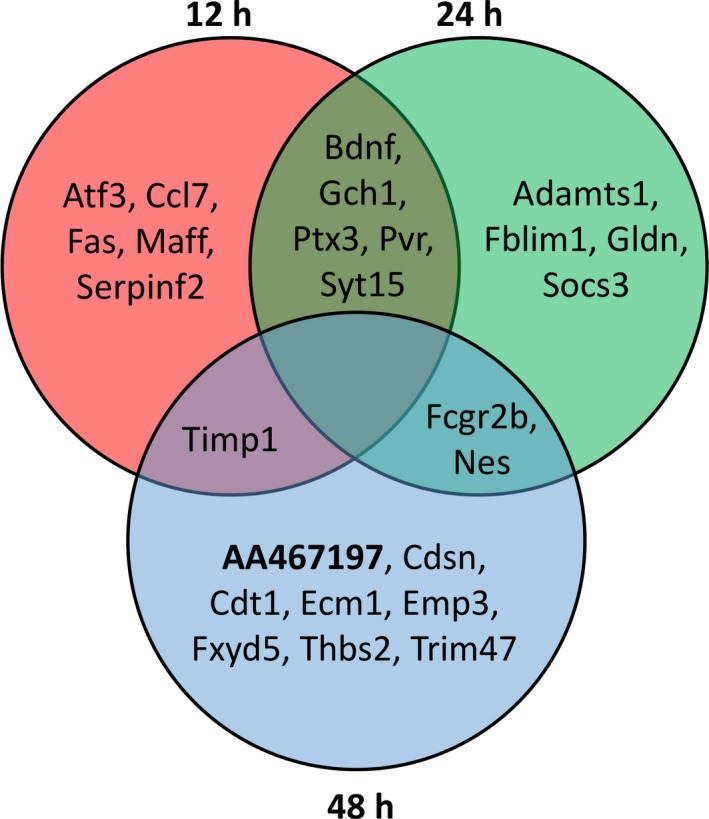
Coexpression network analysis identifies several influential genes with high centrality measures. The 184 statistically significant, differentially expressed genes (DEGs) identified by microarray analysis of 1‐methyl‐4‐phenyl‐1,2,3,6‐tetrahydropyridine (MPTP) exposure in female aldehyde dehydrogenase 1 family member L1, bacterial artificial chromosome ‐ translating ribosome affinity purification (ALDH1L1 bacTRAP) +/− mice were subjected to novel coexpression network analysis. This analysis identified 25 genes that exhibited high node centrality measures in all three categories (degree, closeness, and eigen). The Venn diagram indicates those nodes that were unique to each time point (12, 24, or 48 h), as well as those that were common across different time points. AA467197 (bold) was the only node that also exhibited high betweeness centrality at all time points.

The canonical and activated pathway analyses reported above rely on known, annotated pathways to determine the significance of expression changes on their function. However, the construction of co‐expression networks based on the expression patterns inherent within the dataset, outside of annotated pathways, has the potential to identify the novel involvement of genes in a biological process, such as the response to a neurotoxicant exposure. Using the constrained dataset of 184 genes, novel co‐expression networks were constructed for every subsampled set of mice at each time point in response to MPTP. The global topological differences were quantified by calculating the intra‐ and intergroup GEDs by comparing subsampled networks within the same time point and with other time points, respectively. The intergroup GEDs were significantly higher than the intragroup GEDs (*p *≤ 0.01) (Supporting Information Table S2), indicating that the structural differences in networks of different time points are significantly higher than in networks of the same time point. The edges that were consistently present in every subsampled network were then used to construct a 100% consensus network for each time point. This resulted in rather large and robust consensus networks at 12 and 24 h consisting of 2322 (edge density = 0.1379) and 2059 (edge density = 0.1223) edges, respectively, and a smaller network of 184 edges (edge density = 0.0109) at 48 h, indicating a rather active response 12 and 24 h after MPTP exposure. These consensus networks were further evaluated for local topological changes and degree, closeness, eigenvector, and betweenness centralities at each time point were calculated for each node of the consensus network. Eleven genes were identified at each time point that were present in the degree, closeness, and eigenvector centralities measures (Fig. 6). In general, these centrality measures quantify the importance of a node in the network in terms of: how well connected a node is to the other nodes in the network (degree); how quickly information could be passed through a node to the other nodes of the network (closeness); and, how well connected a node is to the influential nodes in the network (eigenvector centrality). Because of the relative similarity in these measures of centralities, it is not surprising to exhibit higher degree, closeness, and eigenvector centralities by the same nodes at a time point. However, it is interesting that we found several nodes that were active across multiple time points. Five of the 15 nodes (BDNF, PTX3, PYR, SYT15 and GCH1) were among the most central nodes in both the 12 and 24 h networks in terms of the three centrality measures mentioned above, suggesting a similarity in active mechanisms 12 and 24 h after exposure. Furthermore, FCGR2B and NES had high centrality in the 24‐ and 48‐hr networks, while TIMP1 was the only node with high centrality in the 12‐ and 48‐hr networks. This suggests a gradual shift in the active mechanisms in response to MPTP over a time period of 48 h. In addition to these centralities, the influence of genes in a network was evaluated in terms of betweenness centrality. Nodes with high betweenness centrality are important because of their role as gatekeepers/bridges to connect two parts of a network and alterations in this measure may affect the propagation of information from one part of a network to the other. Similar to other centrality measures, nodes such as MAP3K6, CD44, and HKDC1 were among the nodes with high betweenness centralities in 12‐ and 24‐hour networks, whereas CDT1 was the only node among the high betweenness centrality nodes in 12‐ and 48‐h networks. Interestingly, the transcript AA467197 which encodes microRNA‐147 (miR‐147) showed high betweenness centrality across all time points (Fig. 6, bold).

### Genes altered by MPTP exposure show similar profiles with TMT exposure

**Figure 7:**
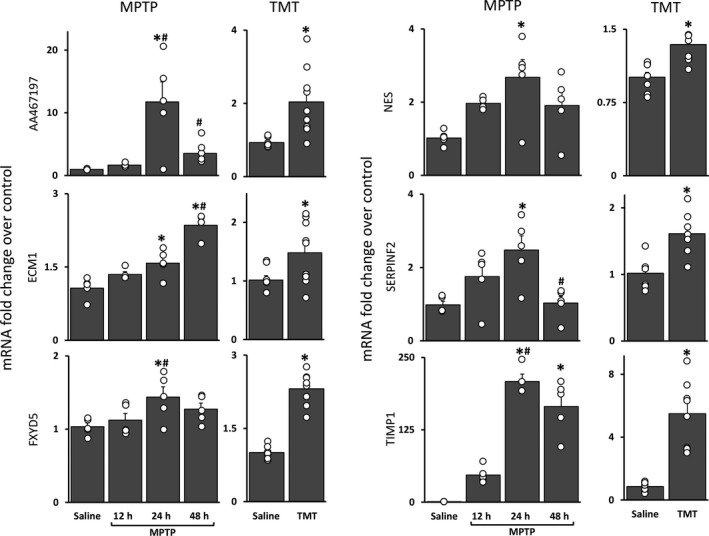
Quantitative PCR verification of genes of interest from microarray analysis using MPTP and TMT. C57BL/6J mice were treated with saline or 1‐methyl‐4‐phenyl‐1,2,3,6‐tetrahydropyridine (MPTP) and Long–Evans rats were given saline or trimethyl tin (TMT). mRNA expression of AA467197, ECM1, FXYD5, NES, SERPINF2, and TIMP1 were measured in striatum [MPTP (12, 24, and 48 h post)] and hippocampus [TMT (5 days post)]. Bars represent mean ± SEM (*N* = 4–9 mice/group) with overlay of individual data points. Statistical significance of at least *p* < 0.05 is denoted as (*) for the neurotoxicant alone groups compared to saline control and (#) in comparison to the previous time point (i.e 12 vs. 24 h, 24 vs. 48 h).

Evaluation of the gene ontology, canonical and activated pathways, and co‐expression network analyses identified several genes of interest/influential nodes that we chose to verify and investigate the expression of across our different neurotoxicant exposures. Because of the expression patterns observed over the MPTP time course and since most of the genes of interest showed expression changes early after exposure, an appropriate ‘early’ time point was chosen for TMT (5 days) to best capture potential expression changes. Of the nine genes evaluated, only six (AA467179, ECM1, FXYD5, NES, SERPINF2, and TIMP1) were significantly affected by both MPTP and TMT exposure at the time points evaluated. While these genes showed a significant increase in expression at least at one time point in the MPTP time course, only ECM1 displayed a similar expression pattern to what was observed by microarray (Fig. 7). Interestingly, the magnitude of expression of TIMP1 was greatly increased over the fold changes observed with the microarray analysis. However, it is unclear whether this is because of the signal compression inherent with microarray analysis or non‐astrocytic expression of TIMP1. Overall, TIMP1 and AA467197 (miR‐147) showed the most significant expression changes across both neurotoxicants (Fig. 7), suggesting that these transcripts may serve as useful biomarkers for neurotoxicant exposure.

## Discussion

As a result of the inherent diversity of cell types in the CNS, it has been difficult to define biomarkers of neurotoxicity that reveal toxicant‐induced damage regardless of the cell type or region affected. However, the induction of astrogliosis in response to neurotoxic insult seems to be a uniform response to damage of all types of cells in the CNS (O’Callaghan and Sriram, 2005). Thus, discovering and characterizing sensitive and specific biomarkers of astrogliosis also could lead to broadly applicable biomarkers of neurotoxicity. In the present study, we demonstrated the usefulness of the ALDH1L1 bacTRAP mouse to discover new potential biomarkers of toxicant‐induced astrogliosis. If the astrocytic gene‐expression profiles we identified following exposure to MPTP can be generalized to other neurotoxic exposures, then these observations may lead to a panel of astrogliosis biomarkers useful for detecting neurotoxicity regardless of the brain region/cell type affected or of the mechanism underlying the effects of a given neurotoxicant.

As neurotoxicant exposure can result in significant astrogliosis, as evidenced by dramatic increases in the expression of GFAP, it was important to ensure that this response would not significantly affect the expression of ALDH1L1. This scenario could potentially disrupt the control of the bacTRAP transgene expression resulting in the over‐expression of the eGFP‐tagged ribosomes and, thus, affecting the TRAP‐ed RNA pool, if not mRNA translation in general. Our results reveal that ALDH1L1 expression remains stable in both the striatum and hippocampus following exposure to MPTP or TMT, respectively. These findings can also be extended to the dopaminergic neurotoxicant, methamphetamine, and the hippocampal neurotoxicant, kainic acid (data not shown). Based on these observations, ALDH1L1‐based control of the bacTRAP transgene seems appropriate for neurotoxicity studies.

Our evaluation of both male and female ALDH1L1 bacTRAP mice revealed several interesting genotype‐associated alterations in the response to MPTP exposure. We found that astrocyte hypertrophy, measured by striatal GFAP concentration, was augmented in +/‐ and +/+ male and female ALDH1L1 bacTRAP mice. Interestingly, baseline GFAP levels were also elevated in these animals. This increase in GFAP protein concentration requires further investigation as the cause remains unclear. Additionally, we showed that male +/+ and all female ALDH1L1 bacTRAP mice seem to have an enhanced protection from MPTP‐induced TH and DA loss. Specifically, +/+ females showed very little TH loss and no significant DA loss following MPTP exposure (Fig. 4). Presently, we do not understand the impact of this finding. Generally, females have more complexity within their immune system and higher circulating levels of estrogen (Yeretssian *et al.,* 2009; Klein and Flanagan, 2016) and it is possible that these characteristics may influence the response of female ALDH1L1 bacTRAP mice compared to C57BL/6J counterparts. However, further investigation into the potential causes must be examined and implications may provide valuable insight into the mechanism of dopaminergic neurotoxicity in this model. Overall, these observations led to our use of female +/‐ ALDH1L1 bacTRAP mice for transcriptomic analysis, as this cohort of animals seemed the least affected by inclusion of the BAC transgene, particularly with respect to GFAP expression.

In an effort to identify new, early biomarkers of neurotoxicity‐driven astrogliosis, we evaluated the astrocyte transcriptome at 12, 24, and 48 h following MPTP exposure; that is, time points that precede the 48‐ to 72‐h window for the peak increase in GFAP (O’Callaghan *et al.,* 1990; O’Callaghan *et al.,* 2014). Genomic analysis of actively translating mRNA in astrocytes identified 184 transcripts whose expression was significantly affected by MPTP exposure. In agreement with our previous findings (e.g. O’Callaghan *et al.,* 2014), we have shown that the majority of the altered genes show peak expression at 24 h post‐MPTP exposure. Using a FACS method, Zamanian *et al. *(2012) also evaluated the astrocyte‐specific response to different neural insults, including stroke and severe neuroinflammation. Interestingly, when comparing the two datasets, we find that only 35 genes are common between both methods. While there is fidelity between the two generated cell‐type‐specific datasets, their divergence highlights the differences between the two applied methods.

Though we have not directly compared the generated transcriptomes following different methods of isolating astrocyte‐specific responses to neurotoxicant exposure, the benefits and limitations of the bacTRAP method compared to other means of cell‐specific evaluation have been discussed previously (Emery and Barres, 2008; Heiman *et al.,* 2008; Heiman *et al.,* 2014). First, bacTRAP allows for the rapid isolation of mRNA from whole, ‘intact’ tissue which most efficiently maintains the *in vivo* environment, a condition not met with FACS or immunopanning. This is a distinct advantage over the methods that require tissue dissociation prior to cell sorting which disrupts the *in vivo* cellular/tissue connections prior to downstream analyses and can introduce confounding morphological and molecular changes in the dissociated cells (Heiman *et al.,* 2008). Second, the material isolated by the ‘TRAPing’ technique is specifically ribosome‐bound mRNA representing those transcripts that are being actively translated at the moment of tissue collection, while the FACS and immunopanning techniques facilitate the collection of total mRNA from the sorted cells. This difference can pose both an advantage and a disadvantage; while isolating ribosome‐bound RNA can help to enhance the dataset to only those genes that are directly recruited by an exposure or condition, the limited amount of isolated bound RNA compared to total RNA of a given tissue can require large amounts of tissue from a single animal or sample pooling that can limit or impede subsequent analysis. However, when directly comparing datasets collected from the FACS or bacTRAP methods, Emery and Barres (2008) noted that while there were many similarities, the relative mRNA expression levels following bacTRAP were much higher. Third, our determination that female, heterozygous ALDH1L1 bacTRAP mice are the most suitable for the evaluation of neurotoxicant‐induced astrogliosis can be a limiting factor for neurotoxicity studies that require male animals. Overall, the bacTRAP transgenic mice present a novel and innovative approach to evaluate changes in the transcriptome by allowing for the maintenance of cell‐to‐cell and tissue‐to‐tissue connections up to the time of sample collection, as well as limiting the isolated mRNA to those transcripts most likely to display expression changes in direct response to an exposure or challenge.

In this study, we used two methods of annotated pathway analysis: (i) canonical pathways (KEGG); and (ii) pathway activation. Canonical pathways represent very large and extensive signaling pathways that may be active in a variety of cells and tissues. While the canonical pathways identified in this study do not represent astrocyte‐specific functions, these pathways highlight the general role of astrocytes in immune/inflammatory function following neurotoxicant exposure. By using a more stringent analysis of the activation of discreet signaling pathways, we were able to expand upon the pathways presented by canonical analysis. The pathway activation analysis used here presents an avenue to directly evaluate functional changes in your sample set by comparing the expression values of genes in a particular pathway against the known conditions required for facilitation and activation of that pathway (i.e., increased expression of a gene(s) involved in positive feedback combined with decreased expression in a gene(s) involved in negative feedback leads to activation of the pathway). While there was a significant amount of overlap between the two annotation‐based methods, the pathway activation analysis helps to narrow the focus of the canonical pathways to more specific signaling modules. However, both of these analyses are constrained by overlay with known annotated pathways. To overcome this, we also evaluated the dataset using co‐expression network analysis that is based off of the expression changes in the dataset without reference to annotated pathways. This analysis generated very large networks that allowed for the identification of transcripts that were unique beyond those identified in the canonical and activation analyses. Overall, the methods used here offer a novel way to process and extract functional changes in large datasets (i.e., microarrays and RNA‐seq) that can provide additional distinct and potentially more meaningful expression analysis that goes beyond the commonly used categorization methods, such as gene ontology and canonical pathway analysis.

Following our extensive analyses of the effects of MPTP on the astrocyte transcriptome, we feel that TIMP1 and miR‐147 (AA467197) are the best targets for further investigation as biomarkers of astrogliosis and neurotoxicity, as well as points for potential intervention. We chose TIMP1 because it was steadily increasing over time in our MPTP dataset, was highly up‐regulated post‐ MPTP and TMT exposures, and is known to be found in astrocytes (Zhang *et al.,* 2006; Moore *et al.,* 2011). Induced by inflammatory cytokines, astrocytic TIMP1 inhibits matrix metalloproteinases which degrade extracellular matrixes, including the blood–brain barrier (Vandooren *et al.,* 2014). In a preliminary study using TIMP1 knockout mice, we investigated the GFAP, TH, and DA protein concentrations after MPTP exposure (data not shown). We found reductions in astrocyte hypertrophy and dopaminergic nerve terminal damage in TIMP1 knockouts compared to C57BL/6J mice. This suggests that TIMP1 signaling may be crucially involved in the neurotoxic response to MPTP, supporting the usefulness of the ALDH1L1 bacTRAP transgenic technology in identifying novel astrocyte‐specific gene markers/targets.

To the best of our knowledge, miR‐147 has not previously been associated with astrocytes or neurotoxicant exposure, though it has been identified with cancer and inflammatory processes. This microRNA is predicted to interact with over 600 transcripts; of these, there are several brain‐associated genes with a target score greater than 80 (Wong and Wang, 2015; Wang, 2016). Most interesting is the potential association between miR‐147 and glial maturation factor‐beta, the suppression of which has been shown to protect dopaminergic neurons from MPTP/MPP+ ‐induced damage (Khan *et al.,* 2014; Khan *et al.,* 2015). As miR‐147 would function to suppress translation of glial maturation factor‐beta, it is possible that this microRNA is up‐regulated in astrocytes in order to control the extent of neuronal damage induced by the neurotoxicants, particularly by regulating the release of inflammatory factors and reactive oxygen species (Fan *et al.,* 2018). The evaluation of the roles of TIMP1 and miR‐147 in the astrocyte response to neurotoxic damage will be crucial to future investigations into unifying mechanisms that underlie toxicant‐induced astrogliosis.

By understanding the astrocyte transcriptome, a pathway‐driven approach to reduce astrocyte hypertrophy can be devised. As the understanding of the importance of astrocyte signaling to the pathogenesis of many neurological disorders grows, so does the need for therapeutic interventions aimed to prevent, or reverse, the priming of astrocytes. Due to the complexity of these pathways, a single therapeutic intervention is not likely to suffice for adequate treatment for neurotoxic insults. Additional information about activation of various pathways following exposure to multiple types of neurotoxic pathways will be highly influential for the development of specific multi‐therapy treatments that can be catered to a variety of insults. The use of bacTRAP technology to study, *in vivo*, the actively translating astrocyte transcriptome will aid in the discovery of biomarkers of astrogliosis and targets for intervention of astrocyte‐induced damage following neurotoxic insults.

## Supporting information


**Table S1.** ALDH1L1 bacTRAP mice do not differ in phenotype.
**Table S2.** Intra and inter group graph edit distance (GED) in subsampled networks.
