# Supplementary material for: Astrocyte‐specific transcriptome analysis using the ALDH1L1 bacTRAP mouse reveals novel biomarkers of astrogliosis in response to neurotoxicity
Source: J Neurochem. 2019 Jul 11;150(4):420–40. doi: 10.1111/jnc.14800 (PMC6771645; doi:10.1111/jnc.14800)
Supplement: Supplementary file 1 — Table S1. ALDH1L1 bacTRAP mice do not differ in phenotype. Table S2. Intra and inter group graph edit distance (GED) in subsampled networks. [file JNC-150-420-s001.pdf]

Title: Astrocyte-specific transcriptome analysis using the ALDH1L1 bacTRAP mouse reveals novel biomarkers of astrogliosis in response to neurotoxicity

Running Title: Astrocyte transcriptome and neurotoxicity

Authors: Lindsay T. Michalovicz[1§], Kimberly A. Kelly[1§], Saurabh Vashishtha[2], Rotem Ben-Hamo[3], Sol Efroni[3], Julie V. Miller[1], Alicia R. Locker[1], Kimberly Sullivan[4], Gordon Broderick[2], Diane B. Miller[1], James P. O'Callaghan[1*]

[1]Health Effects Laboratory Division, Centers for Disease Control and Prevention – National Institute for Occupational Safety and Health, Morgantown, WV, USA.

[2]Center for Clinical Systems Biology, Rochester General Hospital Research Institute, Rochester, NY, USA.

[3]The Mina and Everard Goodman Faculty of Life Sciences, Bar-Ilan University, Ramat-Gan 5290002, Israel.

[4]School of Public Health, Boston University, Boston, MA, USA.

[§]These authors contributed equally to the work.

[*]Corresponding Author

Supplemental Table 1: ALDH1L1 bacTRAP mice do not differ in phenotype.

**MALES**	C57 Saline		C57 MPTP		-/- Saline		-/- MPTP		+/- Saline		+/- MPTP		+/+ Saline		+/+ MPTP	
BRAIN:	Ave (g)	Sem	Ave (g)	Sem	Ave (g)	Sem	Ave (g)	Sem	Ave (g)	Sem	Ave (g)	Sem	Ave (g)	Sem	Ave (g)	Sem
Whole brain	0.48	4.9E-03	0.47	1.2E-01	0.47	2.8E-02	0.48	2.3E-03	0.47	4.7E-03	0.45	4.3E-03	0.44	4.7E-03	0.455	4.8E-03
	Ave (%)	Sem	Ave (%)	Sem	Ave (%)	Sem	Ave (%)	Sem	Ave (%)	Sem	Ave (%)	Sem	Ave (%)	Sem	Ave (%)	Sem
Olfac Bulb	0.05	3.1E-03	5.07	1.3E+00	0.05	2.0E-03	5.08	3.6E-03	0.05	2.6E-03	4.76	1.8E-03	0.04	1.1E-03	3.99	9.2E-03
Cerebellum	13.27	9.1E-03	11.84	3.3E+00	12.84	8.9E-03	12.4	1.4E-03	13.14	2.6E-03	12.84	6.9E-03	13.18	5.7E-03	10.95	2.4E-02
Hypothalamus	1.83	1.1E-03	1.82	4.7E-01	1.56	1.0E-03	1.35#	5.5E-04	1.39#	1.3E-03	1.48	6.6E-04	1.58	8.8E-04	1.21#	2.7E-03
Hippocampus	5.2	1.1E-03	6.04	1.5E+00	4.98	2.6E-03	5.55	1.4E-03	5.25	2.6E-03	5.52	2.3E-03	5.25	1.6E-03	4.7	1.1E-02
Striatum	3.58	2.9E-03	3.29	8.9E-01	3.49	2.0E-03	3.61	2.3E-03	3.64	1.7E-03	3.2	3.4E-03	3.62	2.8E-03	2.42*#	5.5E-03
Cortex	37.36	5.0E-03	36.14	9.5E+00	37.12	9.5E-03	37.51	4.1E-03	35.32	7.7E-03	38.25	9.9E-03	34.88	1.6E-02	32.72	7.2E-02
Brain Stem	13.62	4.1E-03	13.72	3.5E+00	13.93	1.1E-02	13.72	5.6E-03	15.3	4.4E-03	14.46	7.4E-03	14.67	9.1E-03	11.63	2.5E-02
Midbrain	13.58	5.5E-03	13.73	3.5E+00	12.32	4.5E-03	13.28	4.4E-03	12.79	5.8E-03	12.53	5.2E-03	11.9	6.9E-03	9.83#	2.2E-02
Pituitary	0.21	2.2E-05	0.21	5.4E-02	0.22	1.4E-04	0.21	1.0E-05	0.21	2.5E-02	0.22	2.2E-05	0.23	2.4E-05	0.18	3.9E-04
BODY:	Ave (g)	Sem	Ave (g)	Sem	Ave (g)	Sem	Ave (g)	Sem	Ave (g)	Sem	Ave (g)	Sem	Ave (g)	Sem	Ave (g)	Sem
Total body	31.86	1.5E+00	30.82	7.7E+00	30.34	4.9E+00	33.68	1.7E+00	35.42	1.4E+00	29.62	2.4E+00	28.3	1.7E+00	30.68	2.3E+00
	Ave (%)	Sem	Ave (%)	Sem	Ave (%)	Sem	Ave (%)	Sem	Ave (%)	Sem	Ave (%)	Sem	Ave (%)	Sem	Ave (%)	Sem
thymus	0.12	9.1E-05	0.09*	2.8E-02	0.17#	2.9E-04	0.13#	3.1E-05	0.13	4.3E-05	0.13#	1.5E-04	0.16#	9.2E-05	0.13#	4.2E-05
spleen	0.25	2.6E-04	0.23	6.3E-02	0.25	1.0E-04	0.27	1.5E-04	0.32#	2.0E-04	0.27	1.9E-04	0.31	2.5E-04	0.29	2.0E-04
liver	3.98	1.8E-03	4.06	1.0E+00	4.36	1.6E-03	4.23	1.4E-03	4.1	9.6E-04	4.55	2.1E-03	4.31	3.3E-03	4.38	2.0E-03
kidneys	1.2	4.8E-04	1.18	3.1E-01	1.01	8.8E-04	1.05	4.2E-04	1	5.0E-04	1.16	8.0E-04	1.1	6.8E-04	1.08	7.2E-04
adrenals	0.01	1.8E-05	0.02	4.2E-03	0.01	1.4E-05	0.02*	9.9E-06	0.02	8.5E-06	0.02	1.5E-05	0.02	8.4E-06	0.02*#	1.3E-05
testes	0.63	1.4E-04	0.69	1.7E-01	0.54	4.9E-04	0.59	3.9E-04	0.54	3.5E-04	0.53#	9.4E-04	0.69	5.0E-04	0.65	5.8E-04

**FEMALES**	C57 Saline		C57 MPTP		-/- Saline		-/- MPTP		+/- Saline		+/- MPTP		+/+ Saline		+/+ MPTP	
BRAIN:	Ave (g)	Sem	Ave (g)	Sem	Ave (g)	Sem	Ave (g)	Sem	Ave (g)	Sem	Ave (g)	Sem	Ave (g)	Sem	Ave (g)	Sem
Whole brain	0.48	6.7E-03	0.48	5.5E-03	0.48	4.5E-03	0.47	6.3E-03	0.47	6.3E-03	0.45*#	1.8E-02	0.46	5.0E-03	0.46	7.9E-03
	Ave (%)	Sem	Ave (%)	Sem	Ave (%)	Sem	Ave (%)	Sem	Ave (%)	Sem	Ave (%)	Sem	Ave (%)	Sem	Ave (%)	Sem
Olfac Bulb	5.39	2.7E-03	5.22	5.0E-03	5.1	3.0E-03	4.94	2.6E-03	4.46	2.8E-03	5.24	4.0E-03	5.03	5.9E-03	5.02	2.0E-03
Cerebellum	12.23	3.5E-03	12.43	3.6E-03	12.61	2.7E-03	12.61	3.5E-03	12.95	3.9E-03	12.21	5.1E-03	12.9	5.8E-03	12.36	6.2E-03
Hypothalamus	1.45	1.1E-03	1.79*	1.6E-03	1.78#	1.0E-03	1.69	1.1E-03	1.78#	8.3E-04	1.57	7.5E-04	2.22#	6.2E-04	1.57*	1.0E-03
Hippocampus	5.43	1.2E-03	5.08	1.2E-03	5.52	2.0E-03	5.32	1.2E-03	5.08	7.6E-04	5.5*#	1.4E-03	5.52	3.4E-04	5.34	1.5E-03
Striatum	2.99	1.3E-03	3.45	1.5E-03	3.56#	2.3E-03	3.32	2.0E-03	3.77#	7.5E-04	3.29*	1.3E-03	3.78#	1.5E-03	3.41	2.1E-03
Cortex	38.11	5.7E-03	36.48	4.4E-03	39.35	5.4E-03	37.25	8.4E-03	40.28	6.6E-03	35.85*	1.2E-02	38.76	1.2E-02	38.6	7.9E-03
Brain Stem	13.75	5.6E-03	13.07	5.2E-03	13.49	2.9E-03	14.08	5.0E-03	13.62	4.6E-03	12.92	5.4E-03	13.78	3.4E-03	14.92	4.1E-03
Midbrain	13.64	4.5E-03	14.13	8.4E-03	14.72	7.9E-03	13.07	6.8E-03	12.97	4.1E-03	16.08*	7.5E-03	12.55	9.3E-03	13.17	5.2E-03
Pituitary	0.21	2.9E-05	0.21	2.4E-05	0.29	5.3E-04	0.29	4.9E-04	0.21	2.9E-05	0.27	4.0E-04	0.22	2.4E-05	0.26	4.3E-04
BODY:	Ave (g)	Sem	Ave (g)	Sem	Ave (g)	Sem	Ave (g)	Sem	Ave (g)	Sem	Ave (g)	Sem	Ave (g)	Sem	Ave (g)	Sem
Total body	23.52	7.2E-01	22.62	8.5E-01	24.1	9.5E-01	27.58#	1.8E+00	23.14	1.2E+00	21.46	2.1E+00	21.4	6.6E-01	21.54	6.1E-01
	Ave (%)	Sem	Ave (%)	Sem	Ave (%)	Sem	Ave (%)	Sem	Ave (%)	Sem	Ave (%)	Sem	Ave (%)	Sem	Ave (%)	Sem
thymus	0.16	1.2E-04	0.15	9.8E-05	0.21#	1.2E-04	0.2#	8.3E-05	0.17	1.3E-04	0.18	5.0E-05	0.19	1.2E-04	0.23*#	2.2E-04
spleen	0.38	2.1E-04	0.38	7.9E-05	0.41	1.5E-04	0.41	3.1E-04	0.4	1.3E-04	0.38	2.4E-04	0.45#	1.7E-04	0.44#	1.8E-04
liver	4.03	2.4E-03	4.47	1.2E-03	4.54#	2.1E-03	4.08	1.3E-03	4.89#	1.8E-03	4.49	1.4E-03	4.67#	1.7E-03	4.4	1.6E-03
kidneys	1.17	3.8E-04	1.21	1.7E-04	1#	3.0E-04	0.96#	6.0E-04	1.09	5.2E-04	1.03#	1.8E-04	1.19	2.6E-04	1.14	5.2E-04
adrenals	0.03	1.9E-05	0.03	1.4E-05	0.03	2.3E-05	0.03	3.7E-05	0.03	2.0E-05	0.03	4.7E-05	0.03	1.4E-05	0.03	2.4E-05
uterus	0.37	9.4E-04	0.35	4.9E-04	0.2	2.8E-04	0.25*#	6.6E-04	0.36	6.3E-04	0.27#	7.9E-04	0.27	2.8E-04	0.25	3.3E-04
2 ovaries	0.02	3.9E-05	0.02	1.7E-05	0.03	4.3E-05	0.05	2.7E-05	0.03	3.6E-05	0.04	8.5E-05	0.03	3.9E-05	0.03	4.0E-05

* $p \leq 0.05$ compared to saline # $p \leq 0.05$ between genotype

Supplemental Table 2. Intra and inter group graph edit distance (GED) in subsampled networks.

Intra Group differences		Inter Group differences		
Compared Networks	Intra Group GED	Compared networks	Inter Group GED	P-value*
Networks at 12 hrs	59.32	12hrs/24 hrs networks	74.74	***0.000***
Networks at 24 hrs	58.12	24 hrs/48 hrs networks	73.05	***0.012***
Networks at 48 hrs	32.95	12hrs/48hrs networks	75.58	***0.000***

*calculated by comparing the median intra and inter group GEDs with Wilcoxon's Ranksum Test

Supplemental Methods

Node degree centrality. The node degree of a node *i* is a measure of connectedness of a node. Unweighted degree centrality is the number of edges linked to node *i*. Weighted node degree centralities are calculated by using the weight of each edge incoming or outgoing from a node.

Closeness centrality. Closeness centrality of a node is the inverse sum of the shortest path length from a node to all other nodes of the network and represents the importance of a node in context of information processing. It is a measure of how fast information spreads from a given node to other reachable nodes in the network (Newman, 2003) and can be calculated as follows:

$$c(i) = \left(\frac{A_i}{N-1}\right)^2 \frac{1}{C_i} \quad (1),$$

where A_i is the number of reachable nodes from node *i*, *N* is the number of nodes in the network and C_i is the sum of path lengths from node i to all reachable nodes. In directed networks, closeness is represented as *'Incloseness'* and *'Outcloseness'*. However, here only weighted closeness centrality scores were calculated for this undirected network.

Node eigenvector centrality

Eigenvector centrality defines the influence of a node in a network by assigning relative scores to all nodes in the network based on the concept that connections to high-scoring nodes contribute more to the score of the node in question than equal connections to low-scoring nodes.

Node betweenness centrality. Node betweenness centrality for each node of inferred networks was calculated using Brandes algorithm (Brandes, 2001). Betweenness centrality of a node *n* reflects the amount of control that this node employs over the interactions of other nodes in the network (Yoon et al. 2006) and can be computed as follows:

$$C_b(n) = \sum_{s \neq n \neq t} \left(\sigma_{st}(n) / \sigma_{st} \right) \qquad (2),$$

where *s* and *t* are nodes in the network different from *n*, σ_{st} denotes the number of shortest paths from *s* to *t*, and $\sigma_{st}(n)$ is the number of shortest paths from *s* to *t* that *n* lies on. Weighted *betweenness* were calculated for each node of the network. Note that, *betweenness* centrality scores were normalized for every node as $C_b(n)/(N-1).(N-2)$.

References

Brandes, U. (2001) A faster algorithm for betweenness centrality, *J Math Sociol.* **25**, 163-177.

Yoon, J., Blumer, A., Lee, K. (2006) An algorithm for modularity analysis of directed and weighted biological networks based on edge-betweenness centrality, *Bioinformatics.* **22**, 3106-3108.

Newman, M. (2003) The Structure and Function of Complex Networks, *SIAM Rev.* **45**, 167-256.
